# Gratitude as an antidote to materialism in young consumers

**DOI:** 10.3389/fpsyg.2024.1352729

**Published:** 2024-04-15

**Authors:** Suzana Valente Battistella-Lima, Tânia Modesto Veludo-de-Oliveira

**Affiliations:** ^1^Saint Paul Business School, São Paulo, Brazil; ^2^FGV EAESP – Escola de Administração de Empresas de São Paulo da Fundação Getulio Vargas, São Paulo, Brazil

**Keywords:** gratitude, materialism, young consumers, intervention, mental health, wellbeing

## Abstract

**Introduction:**

Materialism has consistently been associated with mental health problems, such as depression and anxiety, and with a decline in overall wellbeing. This article seeks to deepen the understanding of whether or not the level of materialism is reduced when a sense of gratitude is instilled and, if so, how.

**Methods:**

After a thorough literature review, two empirical studies are presented. Study 1 has a quasi-experimental design and a sample of adolescent individuals in a major city in Brazil. Study 2 takes the form of a survey, completed by parents, in the same city.

**Results:**

Study 1 demonstrates that a gratitude-based intervention can reduce the subjects’ belief that material wealth brings happiness and signifies success. Study 2 suggests that parents who express gratitude have a perception of raising less materialistic children.

**Discussion:**

These conclusions add to the theory and practice of consumer psychology and responsible consumption, particularly in relation to the behavior of young people, pointing toward ways to reduce excessive consumption through a simple and easily applied intervention: the stimulus of gratitude.

## 1 Introduction

*“There is so much to be grateful for, just open your eyes.” (Anon.)*

Materialism, defined as “the importance a consumer attaches to his worldly possessions” (Belk, 1984, p. 291) and as “a set of central beliefs about the importance of possessions in the individual’s life” (Richins and Dawson, 1992, p. 308), has been consistently linked to mental health issues and a decline in overall wellbeing. Several studies have associated materialism with depression (Mueller et al., 2011; Otero-López and Villardefrancos, 2013; Muñiz-Velázquez et al., 2017; Wang et al., 2017, 2020; Teng et al., 2022) and anxiety (Otero-López and Villardefrancos, 2013; Teng et al., 2022). Furthermore, materialism has been demonstrated to significantly contribute to the prevalence of addictive behaviors, including compulsive buying (Mueller et al., 2011; Otero-López and Villardefrancos, 2013; Harnish et al., 2019), gambling addiction (Burroughs and Rindfleisch, 2012), and addiction to social networking sites (Wang et al., 2020). Overall, materialism exhibits a negative association with subjective wellbeing (Burroughs and Rindfleisch, 2002; Wang et al., 2017) and satisfaction with life (Mueller et al., 2011; Muñiz-Velázquez et al., 2017). Individuals exhibiting materialistic tendencies often experience greater loneliness (Pieters, 2013) and lower self-esteem (Tsang et al., 2014; Nairn and Opree, 2021).

Initiatives discouraging materialism might thus be undertaken to promote mental health and wellbeing by encouraging individuals to diminish the importance attached to their possessions and thereby encourage them to avoid excessive consumption. It is logical to argue that initiatives such as those would be potentially effective if tailored to young people during their development into consumers. One possible strategy for achieving that aim could be to instill a sense of gratitude, which is defined by Emmons and McCullough (2004, p. 9) as “the recognition and appreciation of an altruistic gift,” in those future consumers or even in such key referent individuals in their ambit as their parents. It is commonly asserted that children and adolescents have become increasingly market-mature (Valkenburg and Cantor, 2001), potential influencers of family shopping patterns (Nørgaard et al., 2007) and more materialistic than previous generations (Schor and Henderson, 2008). Compared with their less materialistic counterparts, materialistic young people apply more pressure on their parents in the purchase process on average and specifically think that more should be spent on their birthday and Christmas gifts (Goldberg et al., 2003).

The existence of a sense of purpose in life has been identified as an intermediary factor in mitigating the adverse relationship between materialism and mental wellbeing (Aruta, 2023). It has been suggested that public policies aimed at reducing children’s and adolescents’ materialism—for example by limiting or regulating exposure to marketing communications—have in practice failed to achieve the expected reduction because “they do not address the underlying insecurities that give rise to excess consumption behaviors” (Burroughs et al., 2013, p. 21). Alternative strategies to help children and adolescents resist the rise of materialism in contemporary society must therefore be sought by parents and educators concerned with this issue, such as a focus on rearing socially secure children (Burroughs et al., 2013). It is known that children’s emotional health is enhanced when they express gratitude (Emmons and Shelton, 2002). That quality could be considered as an antidote to materialism (Tsang et al., 2014) since grateful individuals do not prioritize material success as a key factor in the experience of happiness (McCullough et al., 2002).

Two complementary empirical studies will be presented in this paper. The objective of the first study is to provide a better understanding on the causal relationship between gratitude and materialism and its effects on young consumers. The quasi-experimental design of Study 1 was built especially on that of a study by Chaplin et al. (2018), to test whether or not manipulated experimental conditions of low effort and ease of expressing gratitude would diminish materialism. It differs from their study, however, in so far as materialism was treated there as a unidimensional construct whereas Study 1 explores it more broadly as a multidimensional construct, composed of centrality, happiness, and success (Richins and Dawson, 1992). The objective of Study 2 was to elucidate the relationship between parents’ disposition to express gratitude and perception of children’s level of materialism, if any, and to test the extent to which that disposition in the parents resulted in less materialistic children. Considering that public policy initiatives to fight materialism have shown poor results so far, Study 2 helps to clarify the role of family and upbringing in discouraging materialism and ensuring responsible consumption.

It has been asserted that most studies of gratitude as a phenomenon carried out in the U.S.A. “have focused on convenience samples of Caucasians from school districts in high socioeconomic status neighborhoods” (Chaplin et al., 2018, p. 7). By contrast, the purposive samples selected for Studies 1 and 2 contain low-income adolescents from a developing country, Brazil, living in families with a monthly family income below the equivalent of US$ 600.00. “Adolescent” is defined by the World Health Organization as an individual between 10 and 19 years of age. It has been found that choosing a desired gift is so important to parents in Brazil that 8% admit to entering into debt to buy the “right” Christmas gift for their children (SPC Brasil, 2018). The excesses detected in childhood consumption behavior in Brazil led to the publication of a formal Resolution relating to advertising and marketing communication directed at children and adolescents by National Council for the Rights of Children and Adolescents (2014). Although it has been seen to diminish extravagance directed at children to some extent, the Resolution is not backed by any type of sanction against those who contravene it, with the result that parents and educators concerned about materialism have to search for alternative ways to cope.

## 2 Theoretical background

### 2.1 Materialism

Research studies have demonstrated an effect on the mental health and wellbeing of consumers who overvalue their material purchases (e.g., Burroughs and Rindfleisch, 2012). The fact that materialists are, on average, less happy than non-materialists can be explained in terms of Gap Theory (Solberg et al., 2004), which asserts that the former have higher expectations of the satisfaction material goods can deliver. Richins (2013) has shown that such expectations cannot be satisfied simply by the purchase of material goods, leading to a decline in positive emotions. In the effort to maintain positivity, materialists continue to crave new purchases and thereby experience chronic dissatisfaction (Tsang et al., 2014).

Richins and Dawson (1992) have identified three dimensions of materialism: centrality, happiness, and success. With regard to the first of the three, it is argued that materialistic individuals place possessions and their acquisition at the center of their life experience. Materialism is thus a way of life in which the consumption of material goods is a goal, informing an individual’s life plans and, ideally, giving meaning to his or her life. About the second dimension, the argument is that materialists see possessions and acquisition as an essential route to satisfaction and wellbeing. As Belk (1984, p. 291) put it: “At high levels of materialism, possessions assume a central role in the individual’s life and are believed to provide the most intense sources of satisfaction or discontent in life.” Though it can be assumed that most individuals seek to be happy, what differentiates the materialist is the pursuit of happiness through the acquisition of material goods rather than by other means, such as personal relationships or professional achievements. Where the third dimension is concerned, materialistic individuals judge their own success and the success of others according to the quantity and quality of accumulated possessions. The value of possessions lies in their ability to project a desired self-image, and materialists judge their success in life by the extent of their ownership of belongings that project the desired image (Richins and Dawson, 1992).

### 2.2 Gratitude

Gratitude has been conceptualized in many ways: as a moral virtue, an attitude, an emotion, a personality trait (Lambert et al., 2009), or a psychological state (Emmons and Shelton, 2002). In general terms, psychology considers gratitude to be an emotion, while philosophy understands it as a virtue, that being a good habit that contributes to a good personal character (Emmons and Shelton, 2002) or moral quality.

It is possible to infer that a person who feels grateful, whether expressing that gratitude overtly or not, is more likely to also feel liked and cared-for by others. Gratitude, as a psychological state, leads to a sense of appreciation of life (Emmons and Shelton, 2002). It may be expressed in relation to other people, which is considered an interpersonal approach (Garg, 2023), but may also be transpersonal, when related to non-human entities, such as nature, the transcendent (Garg, 2023), or God (Emmons and Shelton, 2002).

Gratitude is related to several indicators of wellbeing, such as low levels of depression, positive emotions, perceptions of the meaning of life, and satisfaction with one’s own life (Tsang et al., 2014). It can generate happiness, better physical health, and deeper and more satisfying interpersonal relationships (Emmons and Shelton, 2002). It is correlated to positive reframing, which involves the ability to perceive something previously seen as unfavorable in a positive light (Garg et al., 2023a). Gratitude leads to positive emotions. Acts inspired by gratitude increase social bonds and friendships, and are associated with better levels of life satisfaction (Lambert et al., 2009). Furthermore, transpersonal gratitude increases overall spiritual wellbeing (Garg, 2023).

The fact that a positive emotion can lead to other positive emotions is described by the broaden-and-build theory of positive emotions (Cohn and Fredrickson, 2009). This theory posits that positive emotions lead to broadened mindsets (e.g., novel thoughts, activities, and relationships), which, in turn, lead to enduring personal resources (e.g., social support and resilience). These personal resources contribute to enhanced success, completing a virtuous spiral of positive emotions producing more positive emotions.

Gratitude can be considered an alternative to improve wellbeing in various situations, including organizational contexts. The incorporation of regular gratitude-based interventions within organizations can decrease workplace toxicity (Mahipalan and Garg, 2023), and diminish turnover intentions while promoting a healthy work environment (Garg et al., 2023b). In educational institutions, gratitude can reduce the effect of teasing by enhancing student’s positive emotions (Bansal et al., 2023).

### 2.3 Gratitude and materialism among young consumers

It has been argued by Polak and McCullough (2006) that the relationship between materialism and gratitude can be bi-directional: “although it may be the case that gratitude reduces materialistic strivings, it is also possible that materialistic strivings inhibit gratitude.” (Polak and McCullough, 2006, p. 357). That phenomenon was corroborated in a study of subjects with a mean age of 15 years 8 months by Froh et al. (2011), confirming that gratitude and materialism have opposing associations with wellbeing. While grateful adolescents attained higher GPA scores and were less envious, less depressed, and more satisfied with life, their materialistic counterparts exhibited a lower GPA combined with higher levels of envy and lower life satisfaction. The relationship was clarified somewhat by Lambert et al. (2009) in an experimental study of young people whose median age was 21 years. A causal relationship was found between a state of gratitude and a temporary diminution in materialism. It was concluded that satisfaction with life mediates that relationship and that the stimulation of gratitude leads to lower levels of materialism than in a condition of envy: that is, low gratitude.

It was further found by Chaplin et al. (2008) that those between 8 and 18 years of age who exhibited high levels of materialism had low levels of pro-social behavior (that is, actions aimed at helping others) and that gratitude “softened” that bi-directional relationship by helping the subjects to value others rather than only themselves. Learning to behave pro-socially is an important aspect of social development that begins during childhood (Eisenberg and Mussen, 1989; Hoffman, 2000). Materialism is assumed to interrupt this development by encouraging self-attention and delaying the ability to focus on other people (Chaplin et al., 2008). As a result, children with higher levels of materialism tend to exhibit lower levels of pro-social development.

It is argued by Burroughs et al. (2013) that materialism can be the result of higher-order psychological needs not being met, which suggests that the roots of materialism would be found in the insecurities of childhood. A causal relationship between low self-esteem and materialism has been identified by Chaplin and John (2007). Correlations have also been found between materialism and other factors, such as economic class (Goldberg et al., 2003), parents’ divorce (Rindfleisch and Burroughs, 2004), and children’s exposure to marketing communications (Goldberg et al., 2003). Although causation has not been proved in the last of those relationships, there is strong evidence that such exposure does seem to bring about an increase in materialism, not vice versa. It has been suggested that exposure to advertising can lead children to believe that some brands will increase their happiness and satisfaction with life (Nairn, 2015).

Only two experiments found during a thorough review of the relevant literature have tested whether or not gratitude is the cause of a decrease in materialism. Kasser (2004) “materialistic desires scale” was applied by Lambert et al. (2009) in a study of adults and the “youth materialism scale” (Goldberg et al., 2003) by Chaplin et al. (2018) in another of adolescents aged from 11 to 17. Both experiments confirmed that gratitude provokes a reduction in materialism. In both of those experiments, materialism was treated as a unidimensional construct, measured on a single scale. Our own Study 1 will test whether or not higher levels of gratitude cause a decrease in materialism. Materialism will be measured by administering a multidimensional scale with three main dimensions (centrality, happiness, and success), devised by Richins and Dawson (1992). The questionnaire will be administered to adolescents from 14 to 19 years old.

Study 1 hypothesizes that:

*H1*: A stimulus to increase gratitude will cause a decrease in materialism for all three dimensions of materialism: centrality, happiness, and success.

Materialism in adolescence is related to interpersonal influences, especially from parents and peers (Chaplin and John, 2010). A survey by Goldberg et al. (2003), answered by parents and their children, found that the children of highly materialistic parents were more materialistic than they would otherwise have been. That finding was confirmed by the study by Chaplin and John (2010) referred to above, who considered parents and peers to be sources of emotional support and psychological wellbeing. They found that greater parental support increased adolescents’ self-esteem which in turn decreased the level of their materialism. The same study also found that high-materialism parents had highly materialistic children, a relationship mediated by self-esteem, in the sense that those parents’ adolescent children exhibited lower self-esteem, which in turn increased their materialism (Chaplin and John, 2010).

The results of a study by Hoy et al. (2013), in which children from 9 to 11 years of age and their biological parents individually completed a questionnaire on gratitude, show a small and significant correlation between gratitude expressed by mothers and children, but no relationship in the case of fathers and children. The researchers believed that the difference could be attributed to such “biological contributions” such as gender-linked inheritance patterns, or to environmental factors. It is worth noting that, in the United States, where the study took place, the traditional allocation of childcare responsibilities results in children spending more time with their mothers than with their fathers and thereby having more opportunity to observe and imitate expressions of maternal rather than paternal gratitude. Another possible explanation of the result is that men tend to be more reserved about demonstrations of gratitude than women, who recognize and express it in more explicit ways. If so, male gratitude would be less influential because children are less aware of it (Hoy et al., 2013).

Taking into account that materialistic parents have materialistic children (Chaplin and John, 2010), that grateful mothers have grateful children (Hoy et al., 2013), and that gratitude is negatively related to materialism (McCullough et al., 2002; Polak and McCullough, 2006; Lambert et al., 2009; Froh et al., 2011), the hypothesis tested in our Study 2 is:

*H2*: Parents’ gratitude is negatively related to parents’ perception of children’s materialism.

## 3 Study 1: quasi-experiment

### 3.1 Materials and methods

The pre-test/post-test quasi-experiment with control group chosen as the methodology for Study 1 follows a “design for generalized causal inference” advocated in Shadish et al. (2002). Before-and-after measurements were taken from two subsets of the total sample: a Gratitude Group (the treatment group) and a Routine Group (the comparison group).

The study sample consisted of adolescents enrolled in a Brazilian nonprofit organization, Sinhazinha Meirelles, which seeks to prepare young people from a deprived neighborhood of São Paulo for the job market. They attend in groups of 30–40 people, one being newly recruited each semester. Seventy-two 14–19-year-olds from two different semesters took part in the experiment; 65 completed it.

Study 1 was approved by the “Ethical Compliance Committee” of the Brazilian higher education institute at which this study was conceived and executed. Informed consent forms were signed by parents, and the adolescents’ consent was obtained orally. The Gratitude Group (*n* = 30) was observed in the first semester and the Routine Group (*n* = 35) in the second semester of the same year. Both exhibited similar socio-demographic characteristics: the mean age of the 63% female Gratitude Group was 15.8 years; for the 70% female comparison group, it was 16.2 years. The average wage in the neighborhood from which the sample was drawn, the equivalent of US$ 536.00, compares with a maximum in São Paulo City of US$ 2,717.00 and a minimum of US$ 347.00, and ranks it in 70th place among 90 districts. The sampling frame is thus clearly low-income. Multidimensional materialism was measured on the 15-item scale devised by Richins (2004), which combines the three dimensions of centrality, happiness, and success. It was modified for our research, as shown in Table A1, based on studies that implemented it in other low-income areas in Brazil (Ponchio and Aranha, 2008) and also on interviews with teachers, a group of eight children 10–12 year-olds, one 14 year-old girl and one 15 year-old boy, at the Sinhazinha Meirelles center. Cronbach’s alpha coefficients were calculated for each of the three dimensions, with the following results. For centrality (3 items), *α* = 0.601 before the experimental intervention and *α* = 0.695 after; for happiness (4 items), *α* = 0.705 before and *α* = 0.780 after; for success (4 items) *α* = 0.609 before and *α* = 0.689 after the intervention. Nunnally (1967) and Hair et al. (2010) suggest that an acceptable level of reliability for exploratory research can be as low as 0.6.

The whole quasi-experiment extended over 8 days. On the first day, participants completed the Richins multidimensional materialism scale and identified themselves on the questionnaire. A brief discussion followed, in which the Gratitude Group defined “gratitude” and gave examples of it, while the Routine Group did the same for “routine.” Discussions in both groups lasted 30 min. The Participants were then instructed to keep a daily journal for a week, the format of which was inspired by the “Gratitude Curriculum” (Greater Good Science Center, 2024). Those belonging to the Gratitude Group were invited to write about things that made them feel grateful, such as “Think about a situation when someone did something to help you. What did this person do? How this made you feel?,” or “Write about three things that you feel grateful for.” The journal kept by participants in the Routine Group recorded routine elements of their daily life, by instructing them to “Write three activities that you do every day,” for example, or asking “Which is the color of the clothes you are wearing right now?” On the eighth and final day, after completed journals had been handed in, the Gratitude Group heard the story from “The Giving Tree” by Shel Silverstein, read to them by the researcher, and discussed how gratitude was treated in the story, as recommended by the Gratitude Curriculum. The book shows how, when a person helps someone else, something is given up in order to do so. The Routine Group heard extracts from “The Missing Piece” by the same author and discussed descriptions of routines in the story.

After the reading and discussion activity, both groups repeated the multidimensional materialism scaling exercise. The Gratitude Group learned about routine, to serve as a “counterfactual”: that is, to understand what would have happened if they had not received the gratitude intervention (Shadish et al., 2002). The objective of the routine intervention was to create a condition with the same characteristics as the gratitude condition, except that participants would not feel gratitude. The “routine” theme was chosen for its apparent neutrality in referring to ordinary, day-to-day events. Any observed changes in materialism would therefore be a result of the gratitude intervention, and not due to the presence of the researcher or any other unobserved variable. About US$ 5.00 in local currency was awarded to each student who completed the experiment, to be their contribution to the cost of their graduation party. Though thus not a personal reward payment for participation, it was nevertheless considered to offer an incentive to take part.

### 3.2 Results

To test H1, scores were calculated using the regression method, and a 2 × 2 (time before and after intervention × gratitude or routine) mixed ANOVA was carried out for each materialism dimension.

A significant interaction between time x activity was found for the happiness [*F*(1, 63) = 8.497, *p* = 0.005] and success [*F*(1, 63) = 10.139, *p* = 0.002] dimensions, suggesting that the effect of the intervention on the two was different for adolescents who underwent the gratitude intervention versus those in the routine intervention condition. Planned contrasts were performed to verify if adolescents in the gratitude and routine conditions had the same level of materialism before the intervention activity. It was found that they did with regard to happiness [Mroutine = 0.050 vs. Mgratitude = −0.059, *F*(1, 63) = 0.193, *p* = 0.662] and success [M routine = 0.089 vs. Mgratitude = −0.104, *F*(1, 63) = 0.606, *p* = 0.439]. Following the activities, participants in the gratitude condition had a lower level of materialism than those in the routine condition for both happiness [Mroutine = 0.313 vs. Mgratitude = −0.365, *F*(1, 63) = 8.310, *p* = 0.005] and success [Mroutine = 0.352 vs. Mgratitude = −0.411, *F*(1, 63) = 10.900, *p* = 0.002]. Descriptive statistics are detailed in Table 1. If there are equal groups at pretest (Shadish et al., 2002), as there were in Study 1, the comparison group post-test can be considered as counterfactual inference for the treatment group, and it is therefore possible to infer that the activity stimulating gratitude is the cause of the decrease in the belief that acquisition is a means of achieving happiness and that possessions define personal success.

**Table 1:** Descriptive statistics of Study 1.

Time	Condition	Materialism dimension	Mean	SD	N
Before activity	Gratitude	Happiness	−0.059	1.056	30
		Success	−0.104	0.872	30
		Centrality	−0.292	0.921	30
	Routine	Happiness	0.050	0.961	35
		Success	0.089	1.102	35
		Centrality	0.250	1.009	35
After activity	Gratitude	Happiness	−0.365	0.945	30
		Success	−0.411	0.821	30
		Centrality	−0.434	0.939	30
	Routine	Happiness	0.313	0.949	35
		Success	0.352	1.014	35
		Centrality	0.372	0.905	35

Concerning to the centrality dimension, materialism was not significantly affected by time × activity interaction [*F*(1, 63) = 1.338, *p* = 0.252]. Planned contrast on centrality showed that, before the intervention activity, participants in the gratitude and routine conditions did not exhibit the same level of materialism [Mroutine = 0.250 vs. Mgratitude = −0.292, *F*(1, 63) = 5.053, *p* = 0.028]. Levels of centrality were lower in the Gratitude Group than in the Routine Group. It was further found that, after the activity, adolescents in the gratitude condition maintained a significantly lower level of centrality than those in the routine condition [Mroutine = 0.372 vs. Mgratitude = −0.434, *F*(1, 63) = 12.395, *p* = 0.001]. Descriptive statistics are detailed in Table 1. Both the observed pre-test and post-test means for centrality were lower for the Gratitude Group than for the routine condition group. It is worth highlighting, however, that it is not possible to say whether or not gratitude affects that dimension, given that the pre-test means significantly differ between the groups.

### 3.3 Discussion

The results of Study 1 partially confirm H1 and suggest that materialism is negatively affected by the gratitude stimuli (defining gratitude; giving examples; writing about feelings of gratitude; discussing the text of The Giving Tree) in two out of its three dimensions. Those stimuli brought about a decrease in the success and happiness dimensions of materialism, but their effect on the centrality dimension remained inconclusive. These findings can be interpreted in the light of the studies by McCullough et al. (2002), seeking to understand the nature of gratitude by analyzing the correlation of gratitude with other constructs, among them materialism. A negative correlation was found between gratitude and success (*r* = −0.25, *p* < 0.01), suggesting that grateful people disagree with the idea that material wealth and success go together, and also between gratitude and happiness (*r* = −0.38, *p* < 0.01), suggesting that such wealth is not an important factor in how happy they are. About the centrality dimension of materialism, the negative correlation with gratitude was smaller and not significant (*r* = −0.07, *p* < 0.10).

Therefore, while Study 1 demonstrates that the gratitude stimuli did reduce both the belief that possessions and acquisition are essential for personal happiness and the tendency to judge people’s success by the possessions they have accumulated, it is possible that gratitude may not influence centrality, a lifestyle in which consumption of material goods is considered a goal and is believed to lend meaning to life (Richins and Dawson, 1992).

## 4 Study 2: survey

### 4.1 Materials and methods

In this second element of the research design, a self-completion questionnaire was administered to a sample comprising low-income parents of boys from 10 to 18 years old, with a mean age of 13 years and 7 months. This group was selected because the theory suggests that adolescent boys from low-income families constitute the most materialistic group observed. Specifically, literature has suggested that men tend to be more materialistic than women (Eastman et al., 1997; Kamineni, 2005), adolescence is the age period of life when we are most materialistic (Jaspers and Pieters, 2016), and low-income adolescents tend to display higher levels of materialism than their wealthier counterparts (Chaplin et al., 2014).

The survey took place on the premises of the Centro Educacional Assistencial Profissionalizante (CEAP) in São Paulo, an N.G.O. delivering complementary education to potentially vulnerable young people. That is, the boys attend private or public regular mandatory school during the mornings and attend CEAP during the afternoon as an extracurricular activity. The respondents answered questions relating to gratitude and materialism, either during a parent-teacher meeting at CEAP (57% of the sample) or during a selection process for the institution (43%). Fathers provided 38% of the final total of 845 usable questionnaires, mothers accounted for 51, and 11% were completed by “other guardians,” such as stepmothers, stepfathers, grandfathers or grandmothers. All lived in one neighborhood, in which the average wage was the equivalent of US$ 489.00 (compared with US$ 536.00 for the neighborhood in Study 1). The survey formed part of a larger study with parents of students from deprived neighborhoods in São Paulo.

A gratitude measurement scale was derived from the work of McCullough et al. (2002) and adapted for use in our study. Responses to four statements, shown in Table A2, were collected from a sample comprising 845 fathers, mothers, and other guardians. The gratitude scale was pre-tested by being administered to 9 parents, 2 CEAP teachers, 3 coordinators, and one voluntary worker. A moderate degree of internal consistency was indicated by a Cronbach’s α coefficient of 0.677. To assess parents’ perception of children’s level of materialism, Study 2 employed a nine-item version of the 15-item scale devised by Richins (2004) that was used in Study 1, which is shown in Table A3. Its purpose was to collect parents’ responses to such statements as “My son admires people who own expensive homes, cars, and clothes.” This shortened version of the original scale is recommended by Richins when materialism is measured at a general rather than by dimensions separately. Due to feasibility concerns, parents provided responses regarding their children’s behavior. The validity of questioning parents about children’s behavior has been asserted by Serketich and Dumas (1996). The scale was adapted to the condition of Study 2 based on studies that had already administered it to low-income individuals in Brazil (Ponchio and Aranha, 2008) and was pre-tested by the same interviews as used for the gratitude scale. There was a high degree of internal consistency, confirmed by a Cronbach’s α coefficient of 0.896.

### 4.2 Results

Hierarchical multiple regression was applied to verify the effect of parents’ gratitude on their perception of boys’ materialism, expressly chosen to control for the effects of covariates (Field, 2013), such as the age of a boy and the type of regular mandatory school attended. In the first phase of the regression, the single predictor entered was gratitude, as shown in Table 2. The result was statistically significant [*F*(1, 843) = 19.51, *p* < 0.01], with parents’ gratitude explaining 2.3% of the variance in parents’ perception of boys’ materialism. When boys’ ages and types of school were entered in the second phase, the total variance explained by the model was 3.7% [*F*(3, 841) = 10.66, *p* < 0.01]. The addition of those two variables explained an additional 1.4% of the variance in the perceived materialism [*R*^2^ Change = 0.14; *F*(2, 841) = 6.11; *p* < 0.001]. In the final adjusted model, the three predictor variables were statistically significant, with parents’ gratitude recording a higher value (*β* = −0.16, *p* < 0.001) than boys’ age (*β* = 0.09, *p* < 0.001) or the type of school attended (*β* = 0.07, *p* < 0.05).

**Table 2:** Summary of results of Study 2.

	*B*	*SE B*	*β*	*t*	*R*	*R* ^2^	*ΔR* ^2^
Step 1					0.15	0.023**	
Constant	−0.10	0.03					
Gratitude	−0.15	0.03	−0.15**	−4.42			
Step 2					0.19	0.037**	0.014**
Constant	−0.77	0.24					
Gratitude	−0.16	0.03	−0.16**	−4.45			
Sons’ age	0.05	0.02	0.09**	2.73			
Type of school	0.17	0.08	0.07*	2.08			

*p < 0.05; **p < 0.01.

It is suggested by Duncan et al. (2014) that subgroup replication of whole-sample regression should be used to check the robustness of the results. Study 2 therefore examined three subgroups of respondents: father vs. mother vs. other guardian; CEAP student vs. CEAP applicants; children attending public vs. private schools. Table 3 shows that in all subgroups except “other guardians” (*p* > 0.05, n.s.), parents’ gratitude is negatively related to their perception of boys’ materialism (*p* < 0.05).

**Table 3:** Summary of subgroup results of Study 2.

Subgroup	*β* (gratitude)	*R*^2^ (adjusted)	F score
Respondent			
Father	−0.17**	0.03	10.40**
Mother	−0.11*	0.01	4.97*
Other guardian	−0.14	0.01	1.96
Origin of children			
CEAP student	−0.14**	0.02	9.8**
CEAP applicant	−0.16**	0.02	10.4**
Type of school			
Private	−0.23**	0.05	11.10**
Public	−0.13**	0.01	10.45**

*p < 0.05; **p < 0.01.

### 4.3 Discussion

The results of Study 2 confirm H2 and suggest that both parents’ gratitude (i.e., fathers’ and mothers’ gratitude) is negatively related to parents’ perception of children’s materialism, which contradicts Hoy et al. (2013) who found no relationship between fathers’ and children’s gratitude. Further research is needed to understand the reasons for this inconsistency, but one possible explanation is that the American males who participated in Hoy et al. (2013) study do not communicate their positive feelings to other people and do not speak positively as often Latin American men do (Fernández et al., 2000). Even though the U.S.A. and Latin America exhibit, in general, the same level of verbal expression of joy, the difference in that behavior between men and women in the U.S.A., the former being less explicit than the latter, is higher than in Latin America (Fernández et al., 2000), so Latin American children have opportunities to observe not only their mothers’ explicit expressions of gratitude but also their fathers’, both influencing their own levels of gratitude and subsequently their materialism. Another possible explanation is that Study 2 observed the relationship between parents and sons, while Hoy et al. (2013) did not differentiate their analysis by gender of children. Since it has been asserted that sons tend to be closer than daughters to their fathers (Starrels, 1994), it is more likely that, in a sample containing only sons, the value transmission by fathers will be more evident. On the other hand, the relationship between the gratitude and perceived materialism of “other guardians” was not significant. This could have been because they did not exercise as strong an influence on children’s perceived values as parents did.

Table 3 shows that the variables added for control purposes, age and type of school attended, were also significant but that parents’ gratitude still exercised a higher level of influence. Indeed, the control variables had no influence on gratitude or materialism in our study, yet their inclusion in the design does add to previous studies of materialism. Age differences in materialism were explained by Chaplin and John (2007), who found materialism to be reduced in children aged between 8 and 9, to increase in early adolescence, from 12 to 13 years of age, to decrease during the years of late adolescents, 16–18, but never to return to the lower levels exhibited by younger children. The relationship between age and materialism is mediated, however, by self-esteem. Early adolescents typically have low levels of self-esteem and that is the age at which materialism increases (Chaplin and John, 2007). Study 2 found a small and significant beta coefficient for age predicting perceived materialism, suggesting that growing older increases parents’ perception of materialism in adolescent boys.

To elucidate this finding, an ANOVA was carried out about early adolescence (10–13 years) and late adolescence (14–18 years). The results contradicted Chaplin and John (2007), indicating based on the sample that late adolescents have higher levels of materialism than early adolescents [Mearly = −0.092 vs. Mlate = 0.090, *F*(1, 878) = 7.379, *p* < 0.01]. The increase in adolescents’ materialism observed in Study 2 can be explained by a review of scientific evidence from the development of self-esteem during life, conducted by Robins and Trzesniewski (2005). Based on a meta-analysis of the findings of 86 articles, a cross-sectional study of individuals between the ages of 9 and 90 years old, and a longitudinal study of individuals aged from 25 to 96 years old, a trajectory of self-esteem during life was constructed. Contradicting Chaplin and John (2007), their study presents evidence for a decline in self-esteem from early adolescence (10–13 years old) and late adolescence (14–18 years old), for both genders, which would explain the increase in perceived materialism noticed on Study 2.

The type of school attended by the adolescents in the sample also had a positive relationship with perceived materialism. Young men from private schools were perceived as less materialistic than their peers from public schools. It is possible to infer that this variable is simply a proxy for income, since families in Brazil who are well-off enough to do so tend to send their children to private schools. This finding helps to corroborate the finding by Goldberg et al. (2003) that young people with high levels of materialism tend to come from low-income families.

## 5 General discussion

The literature review and two studies reported in this paper contribute to answering a question posed by Burroughs et al. (2013, p. 25): “What can be done to reduce materialism among children and adolescents?.” Considering the negative impact of material overvaluation on consumers’ mental health and wellbeing, our research findings demonstrated that gratitude is an effective way to reduce materialism. It was found in Study 1 that a young person’s belief that material wealth brings happiness and means success can be mitigated by the stimulation of gratitude. Furthermore, our research findings established the extent to which parents’ demonstrations of gratitude influence the materialistic perceived attitudes of their children. The inference to be drawn from the findings of Study 2 is that families in which gratitude is expressed and experienced can function as agents preventing high levels of materialism. That is, grateful parents spontaneously reduce the perceived levels of materialism of their children.

These research findings reveal that our work contributes to the existing literature in two important ways. First, it advances the theoretical perspective on the causal relationship between gratitude and materialism by reaffirming that gratitude can serve as an antidote to reduce materialism. Unlike previous studies that did not address the elements of materialism separately, we contribute to the existing literature by providing evidence that this antidote works to diminish two elements of materialism (i.e., happiness and success), while centrality, the third element of materialism, remains unaffected. Second, our research contributes to the literature on family dynamics, which explores the impact of family members on each other in terms of behaviors, attitudes, values, and beliefs. Our study enhance the understanding of the negative relationship between the effects of parents’ gratitude on their children’s materialism levels, as perceived by parents.

The findings of our two studies represent an initial look at how parents and educators can counter youth materialism, thereby mitigating potential health consequences that might derive from an overvaluation of material possessions, such as depression, anxiety, or addictive behavior. Simple actions on the part of parents or educators can stimulate gratitude: for example, the keeping of the kind of “gratitude journal” described in Study 1, or the deployment of a “gratitude app” as an aid. Such apps as “Happify or Gratitude Journal 365” allow the user to record thoughts about gratitude, delivering reminders to reflect or write about things they might be grateful for. Parents can seek out books relevant to the expressing and experiencing of gratitude, encourage reading together and elaborate on the stories in discussions of the content. Teachers can do likewise in school with their students. It is recommended by Froh and Bono (2014) that parents express the expectation that their children will express gratitude. That might be done, for example by directly asking them to write a thank-you letter to a grandparent or send a grateful social media message to a relative or a friend, or by meal times and other “family time” to enquire about the best experiences of an adolescent’s day or some favor received from someone else. If families are to some extent religious, prayers of thanksgiving could be a stimulus to remember blessings and thereby enhance the valuing of gratitude. In all such actions, the aim would be to make young people reflect and realize that they have reason to be grateful. Taking into account that parent’s perceptions might mirror their children’s behavior, adults should improve their own levels of expression of gratitude to counter young people’s materialism. They can foster a culture of gratitude by acting as examples. This can be achieved by positively constructed conversation, explicitly thanking young people for their everyday actions, as appropriate, to show them that their effort to improve someone else’s life is valued and appreciated (Froh and Bono, 2014). In terms of feasible public health interventions, community-based workshops or seminars aimed at parents, educators, and young people could provide practical guidance on incorporating gratitude practices into daily life. Topics might include the establishment of gratitude routines, the use of gratitude journals or apps, and ways to communicate gratitude within family and school environments. Additionally, partnerships with mental health professionals could enhance the effectiveness of the intervention by offering expert insights and resources.

It is to be hoped that the practical initiatives discussed above will encourage fellow researchers to explore other feasible ways of reducing materialism and give a lead, at second hand, to some families and communities in their search for a lifestyle characterized by robust mental health and overall wellbeing.

### 5.1 Limitations and future research

Our study is not without limitations. In Study 1, it was not possible to infer the influence of gratitude on the centrality dimension of materialism because the groups investigated did not present the same level of pretest centrality. Further research should investigate this matter, particularly considering that gratitude may not affect centrality in low income families, who experience a sense of insecurity in response to economic deprivation or other difficult conditions they face (Polak and McCullough, 2006). It would be very hard in practice to counteract the perception that the acquisition of material goods is crucial to their lives. Investigation of this phenomenon among families of high socio-economic status could verify whether or not the way of life in which consumption is a central goal is unrelated to gratitude.

Given that parents answered on behalf of their children in Study 2, further studies could seek a more accurate perspective by having children respond on their own account. It might also investigate possible mediators in the relationship of parents’ gratitude to adolescents’ materialism, such as the materialism of the former and the self-esteem of the latter.

## Data availability statement

The raw data supporting the conclusions of this article will be made available by the authors, without undue reservation.

## Author contributions

SB-L: Conceptualization, Data curation, Formal analysis, Investigation, Methodology, Visualization, Writing – original draft, Writing – review & editing. TV-d-O: Conceptualization, Formal analysis, Methodology, Supervision, Visualization, Writing – original draft, Writing – review & editing.

## Appendix

**Table A1:** Materialism Scale adapted to Study 1.

Materialism	Before intervention	After intervention
Success	(*α* = 0.609)	(*α* =0.689)
I admire people who own expensive homes, cars, and clothes.		
Some of the most important achievements in life include acquiring expensive things.		
I like to own things that impress people.		
I pay a lot of attention to the material objects other people own (cellphone, tablet, tennis shoes, etc.).		
Centrality	(*α* = 0.601)	(*α* = 0.695)
I enjoy spending money on expensive things.		
Buying things gives me a lot of pleasure.		
I like a lot of luxury in my life.		
Happiness	(*α* = 0.705)	(*α* = 0.780)
My life would be better if I owned expensive things I do not have.		
I would be happier if I owned more luxury things.		
I’d be happier if I could afford to buy more things.		
It bothers me when I cannot buy all the things I’d like.		

**Table A2:** Gratitude Questionnaire adapted to Study 2.

Gratitude (*α* = 0.677)
I have so much in life to be thankful for.
If I had to list everything that I felt grateful for, it would be a very long list.
When I think about life, I have much to be grateful for.
I am grateful to a wide variety of people.

**Table A3:** Materialism Scale adapted to Study 2.

Materialism (*α* = 0.896)
I can say about my son that he…
…admires people who own expensive homes, cars, and clothes.
… thinks that his life would be better if he owned certain expensive things he does not have.
… enjoys spending money on expensive things.
… thinks that buying things gives him a lot of pleasure.
… likes to own things that impress people.
… would be happier if he could buy more things.
… is bothered when he cannot buy all the things he’d like.
… likes a lot of luxury is his life.
… thinks that spending money is among the most important achievements in life.
